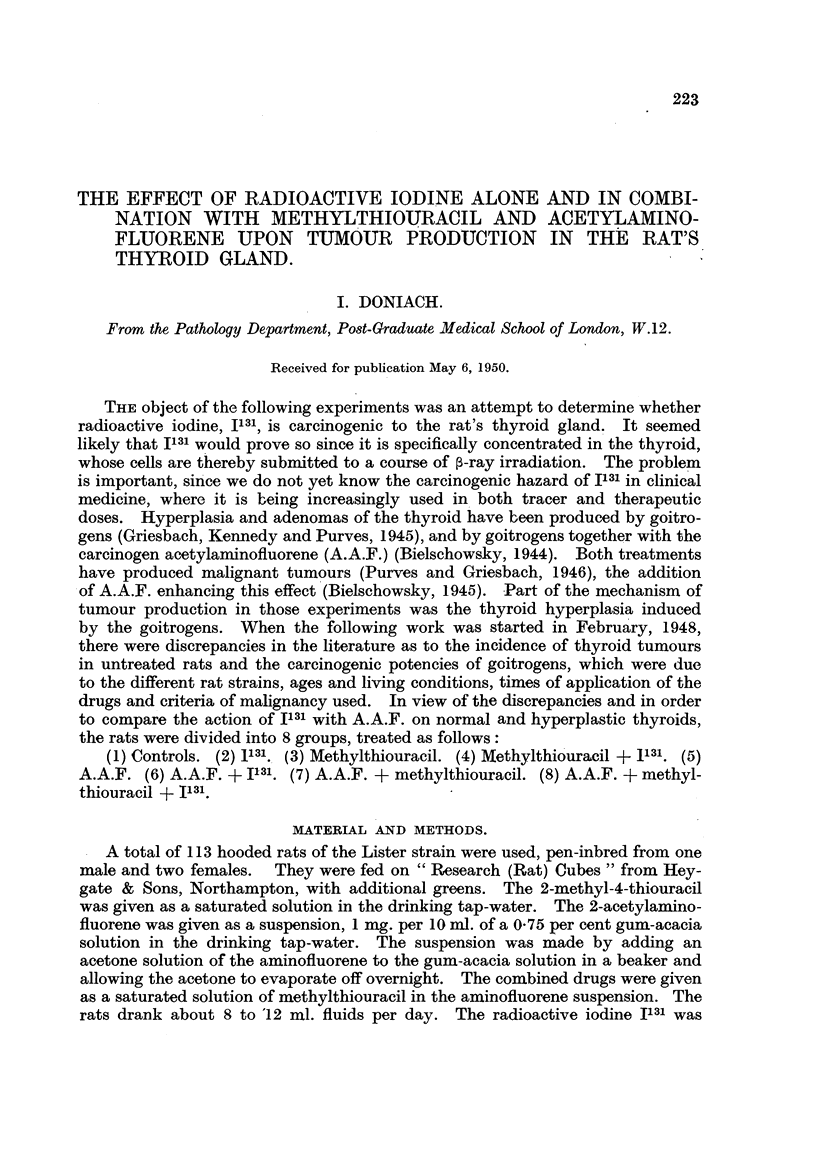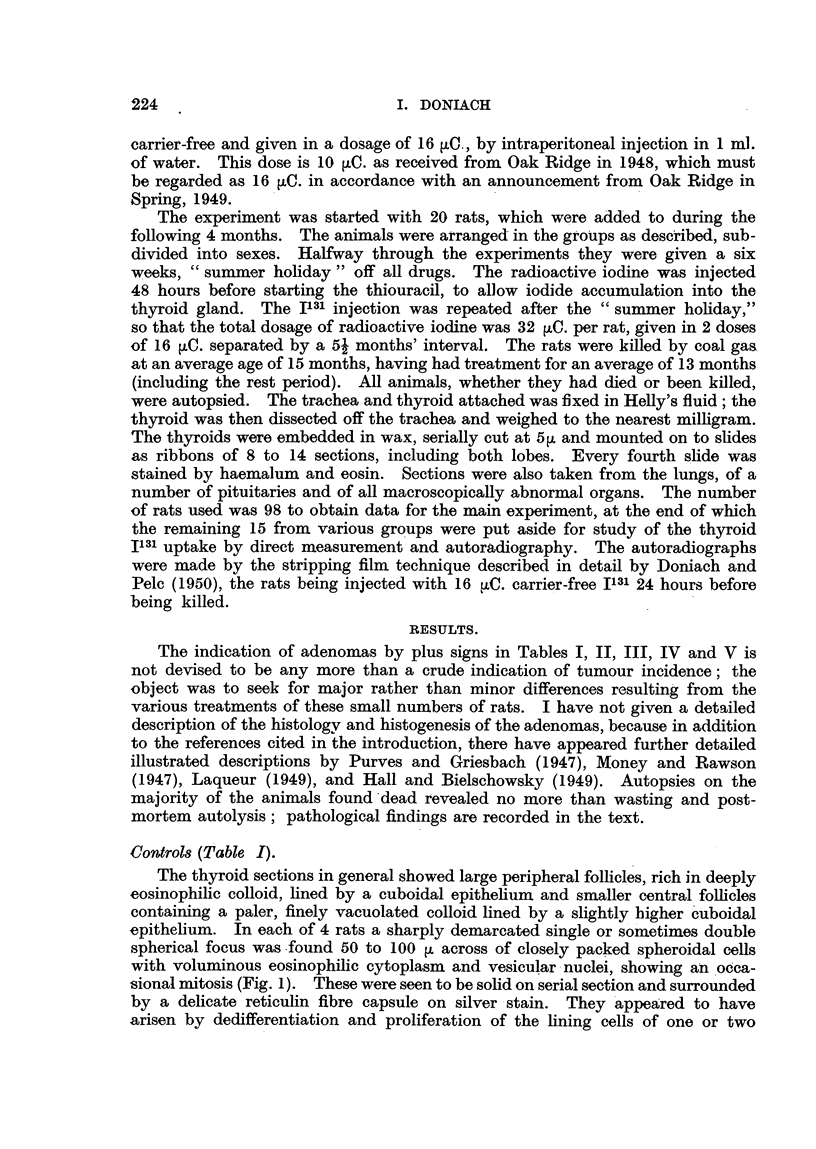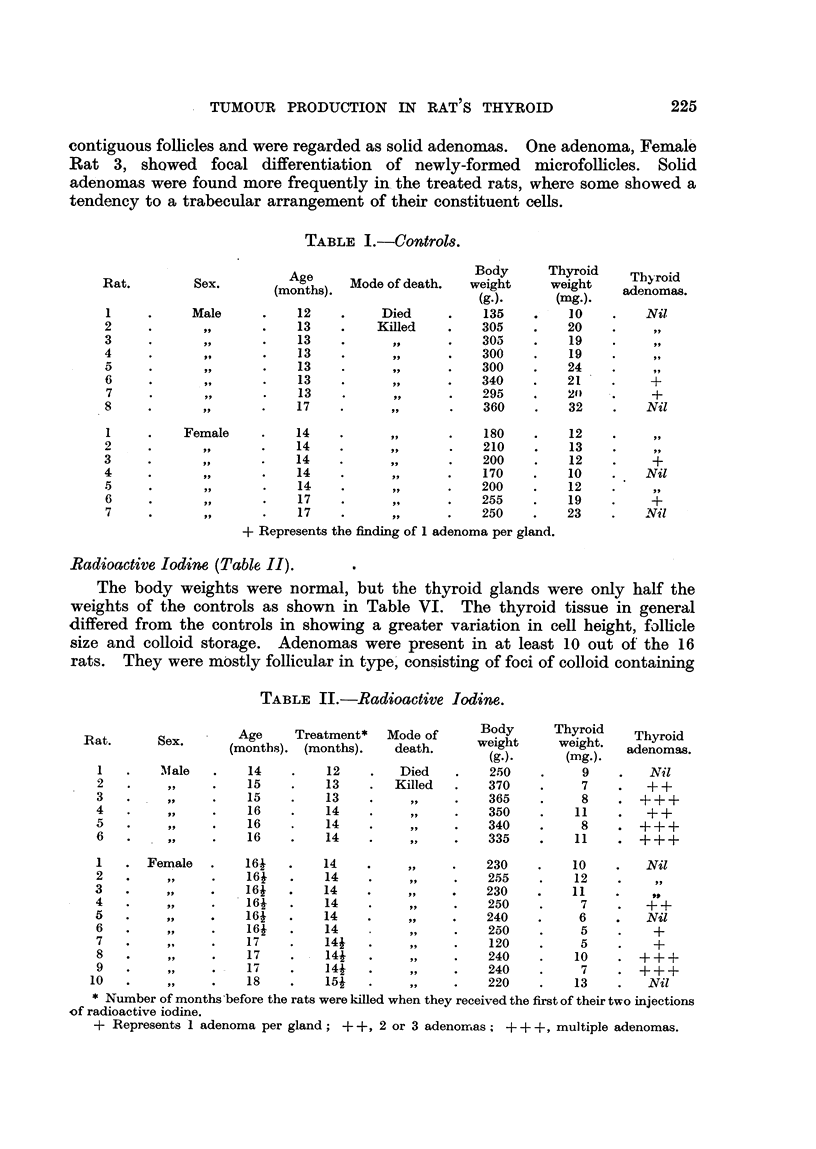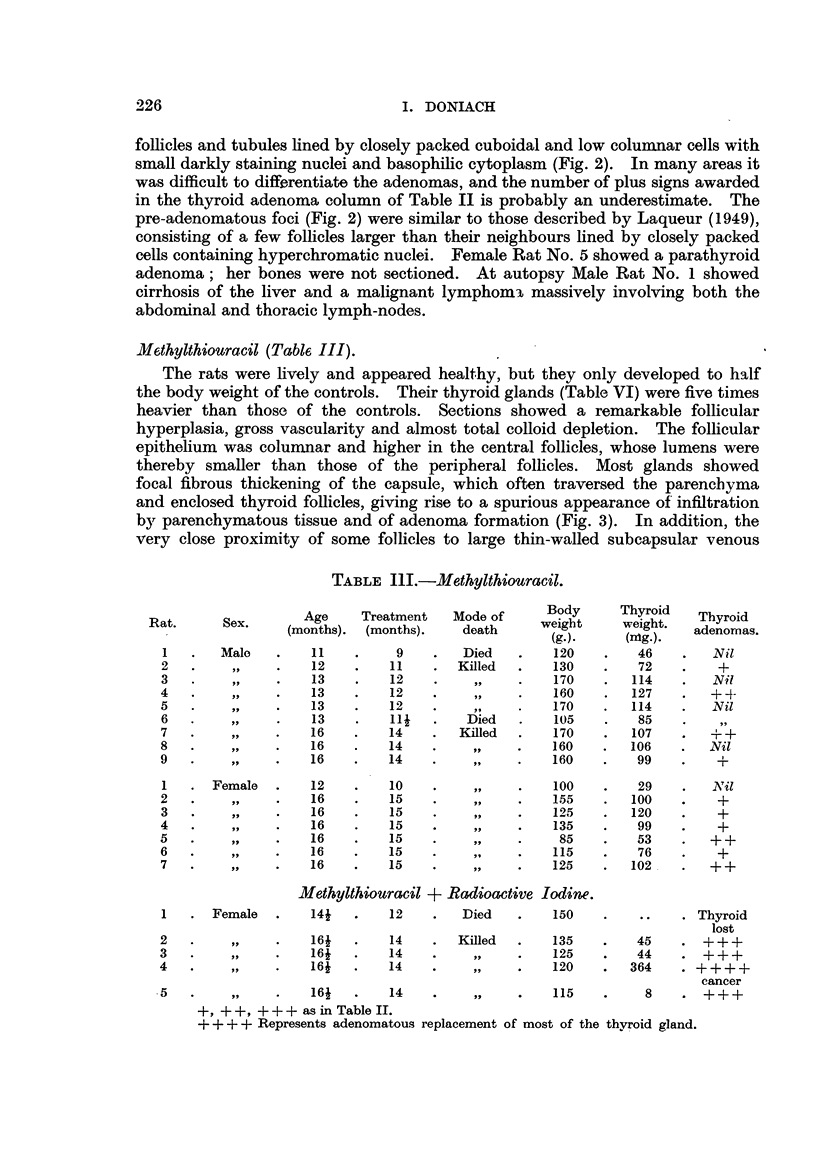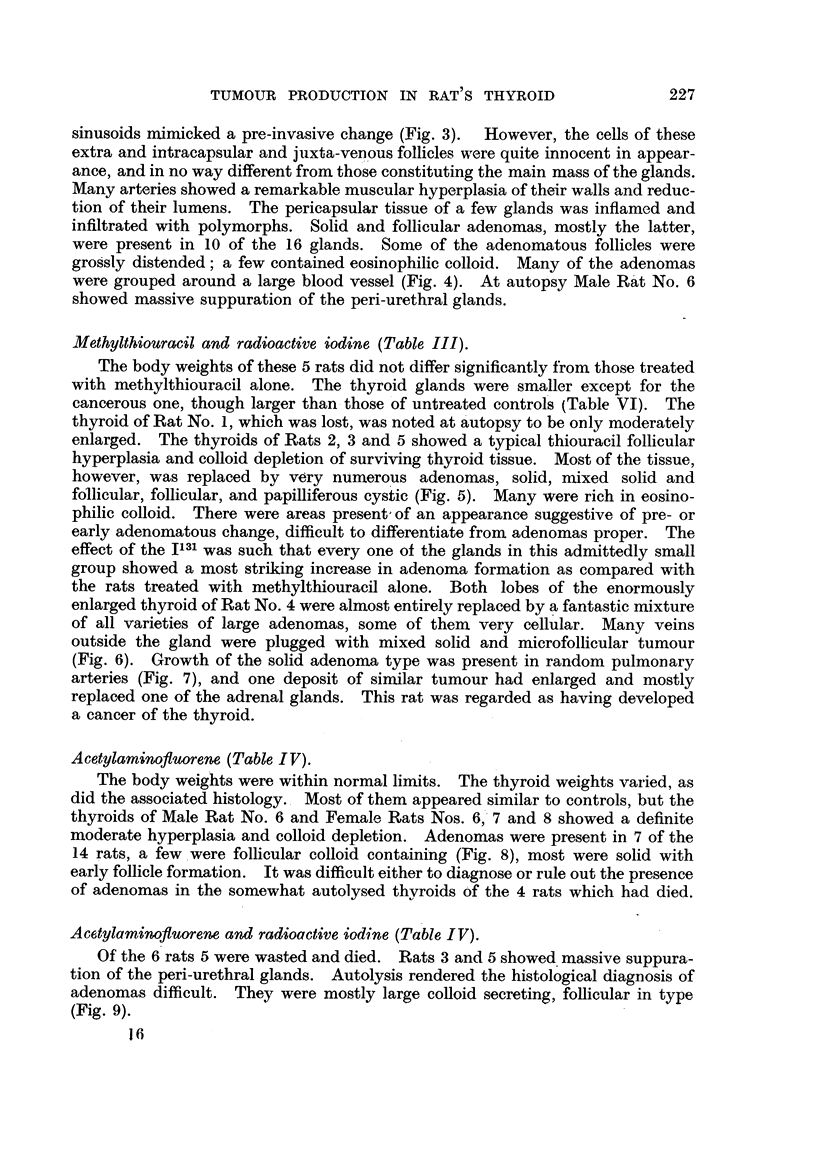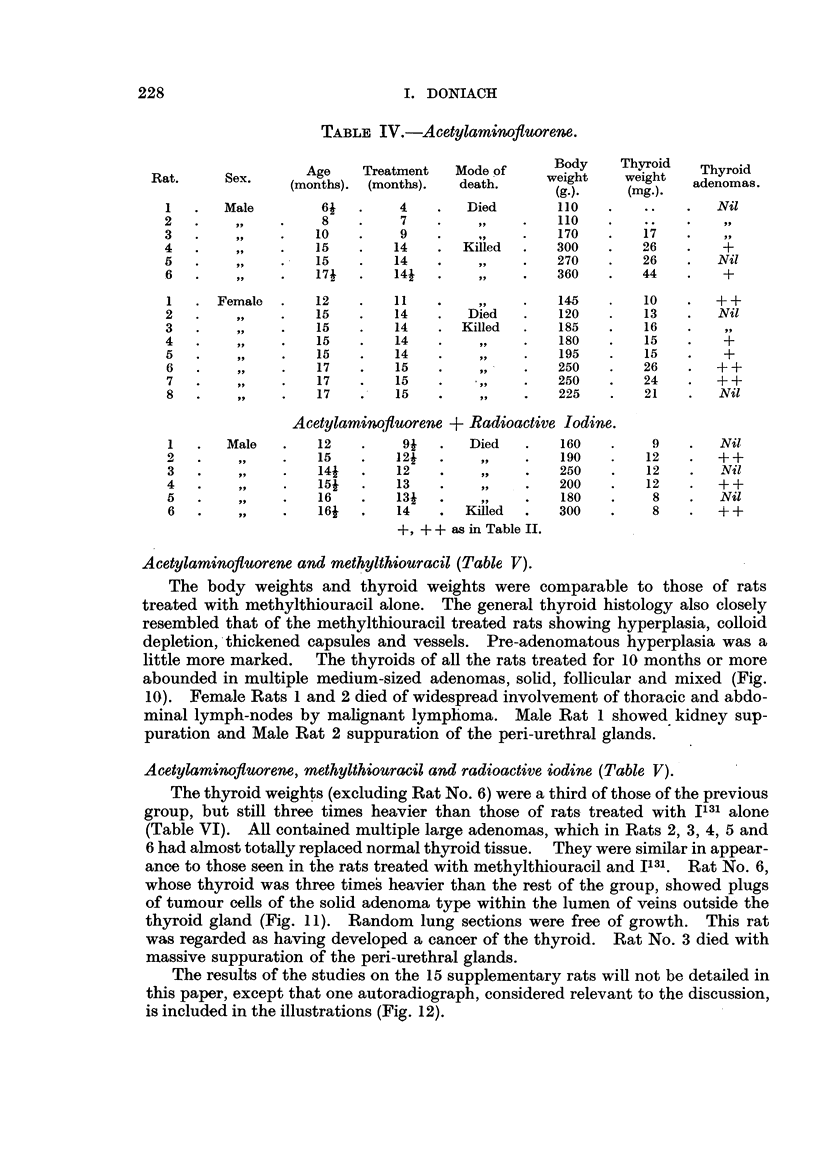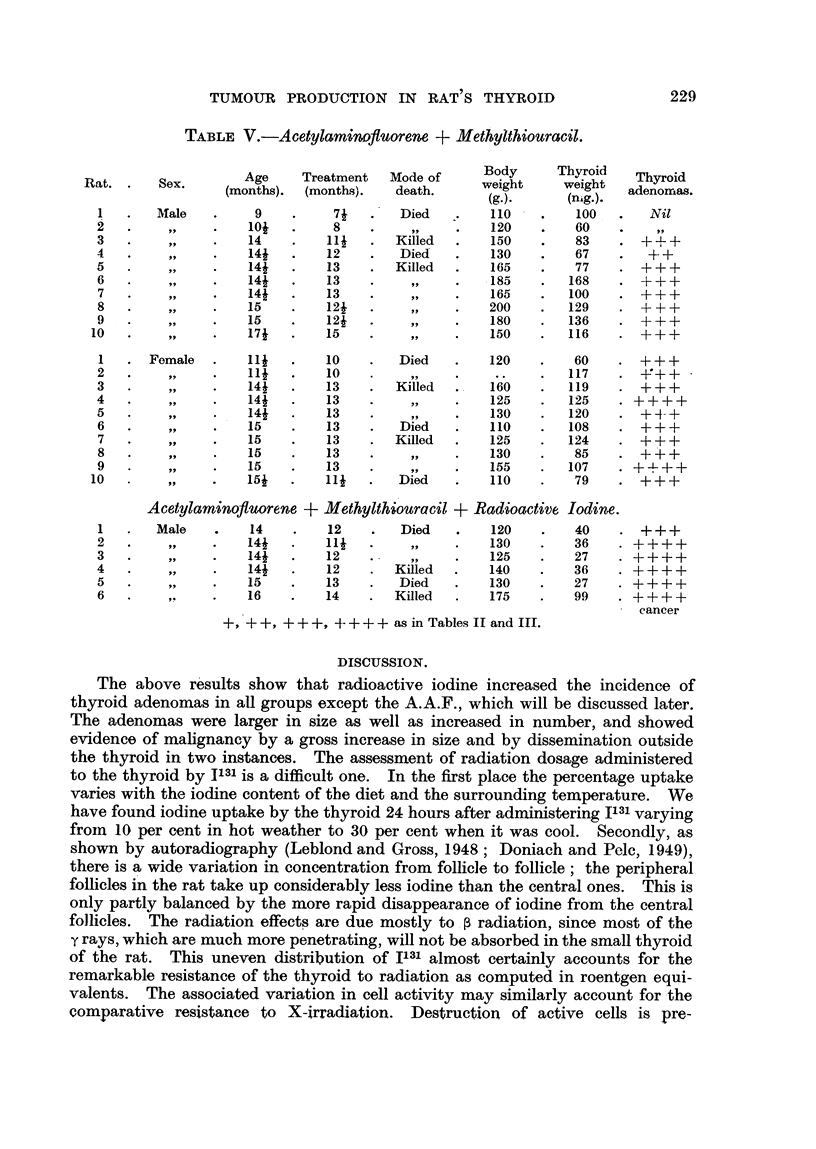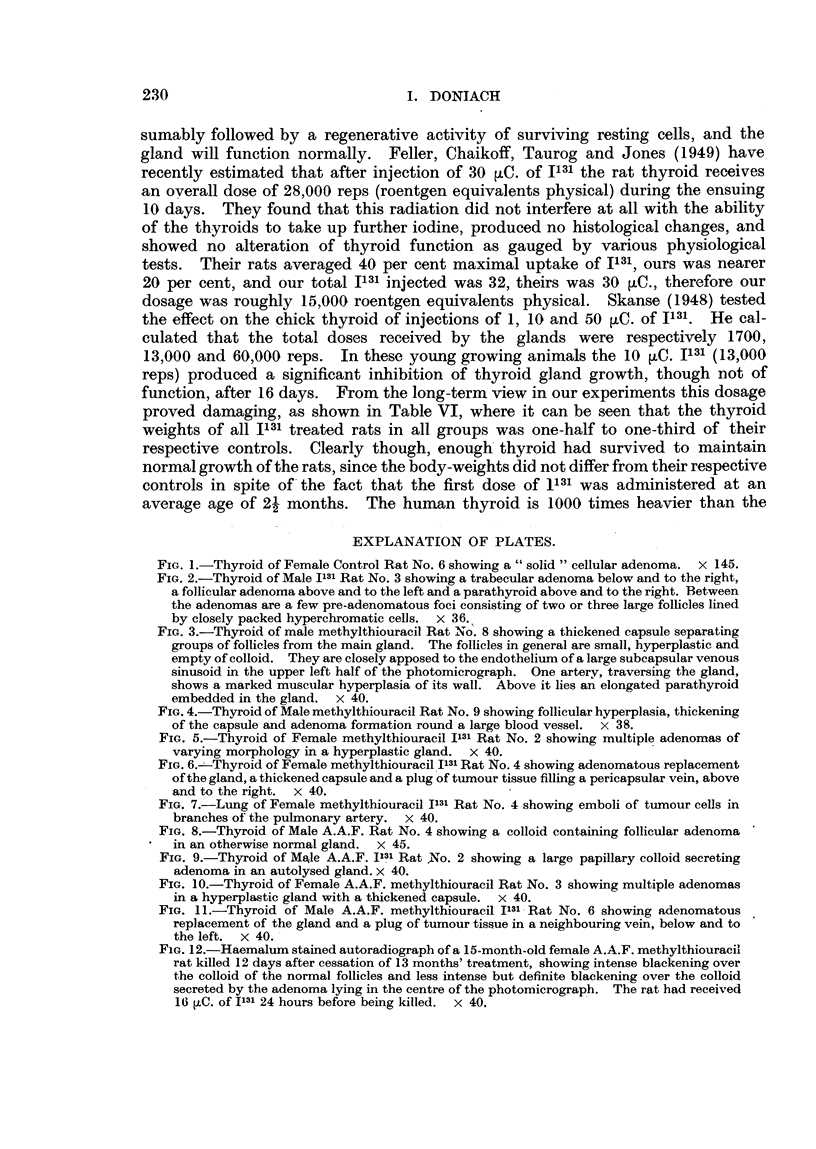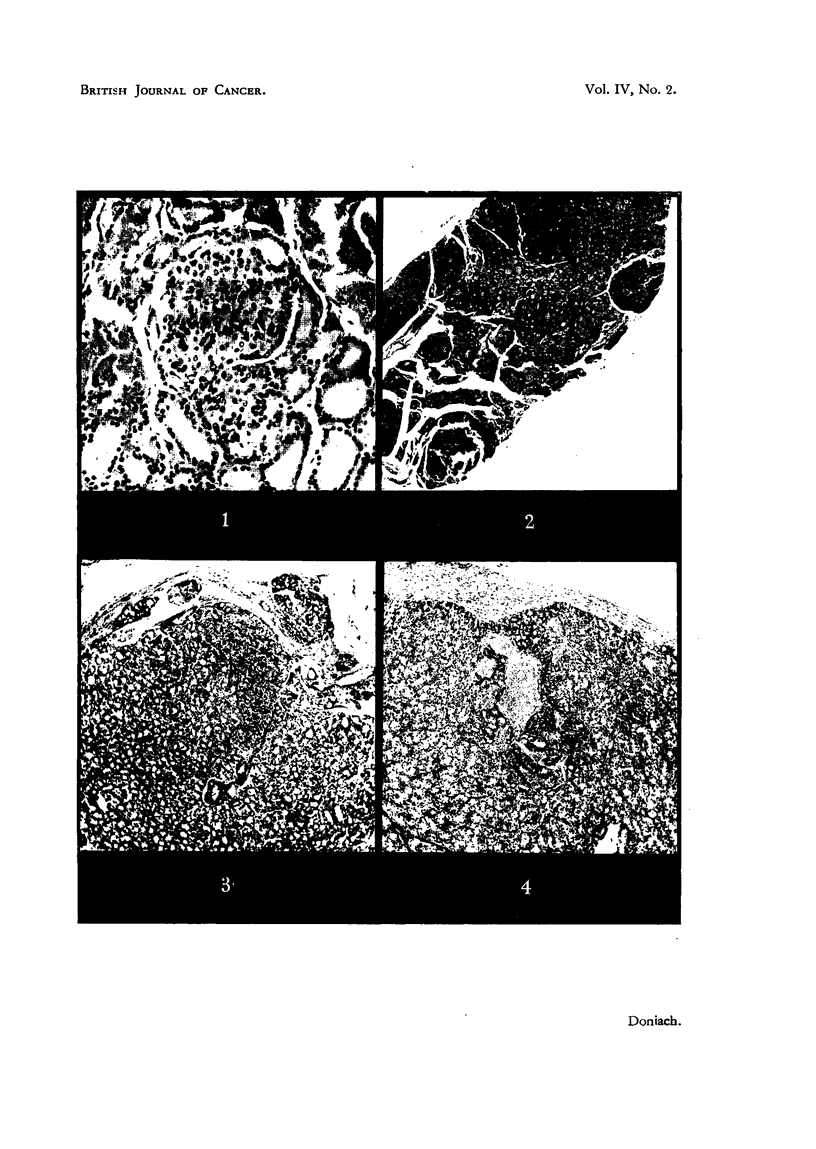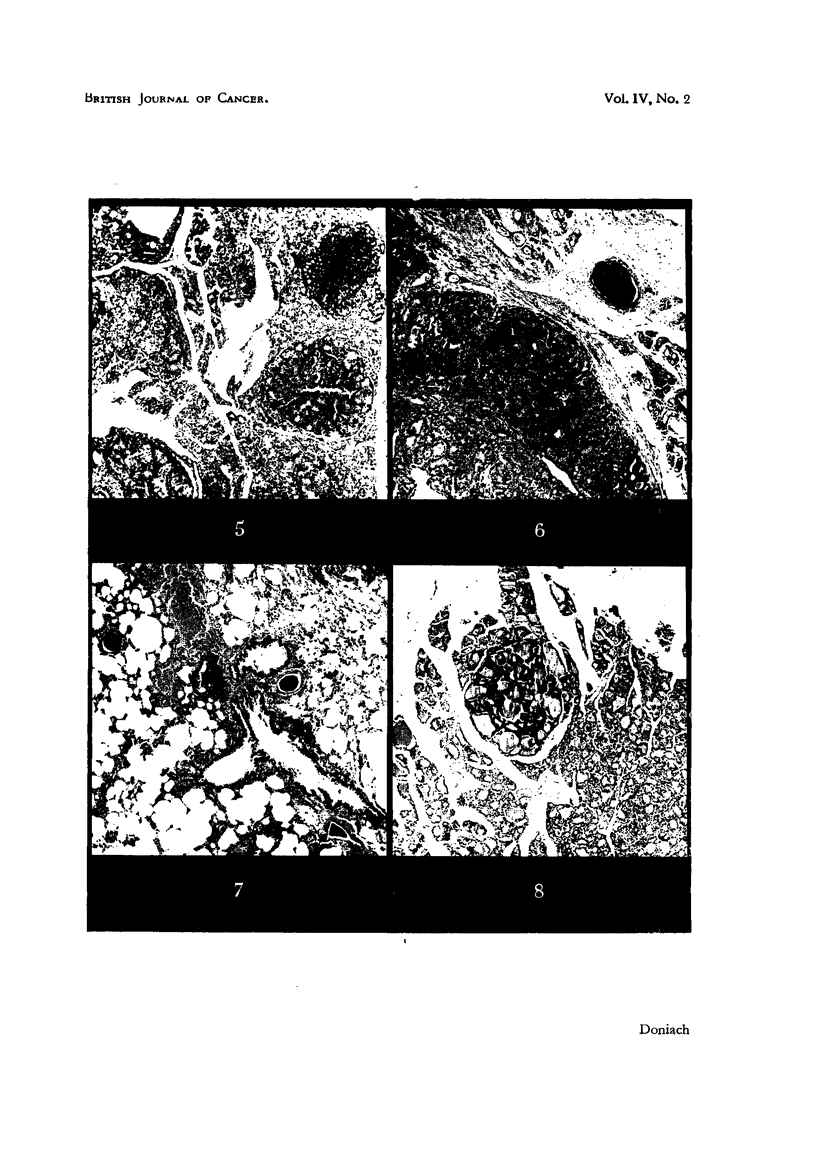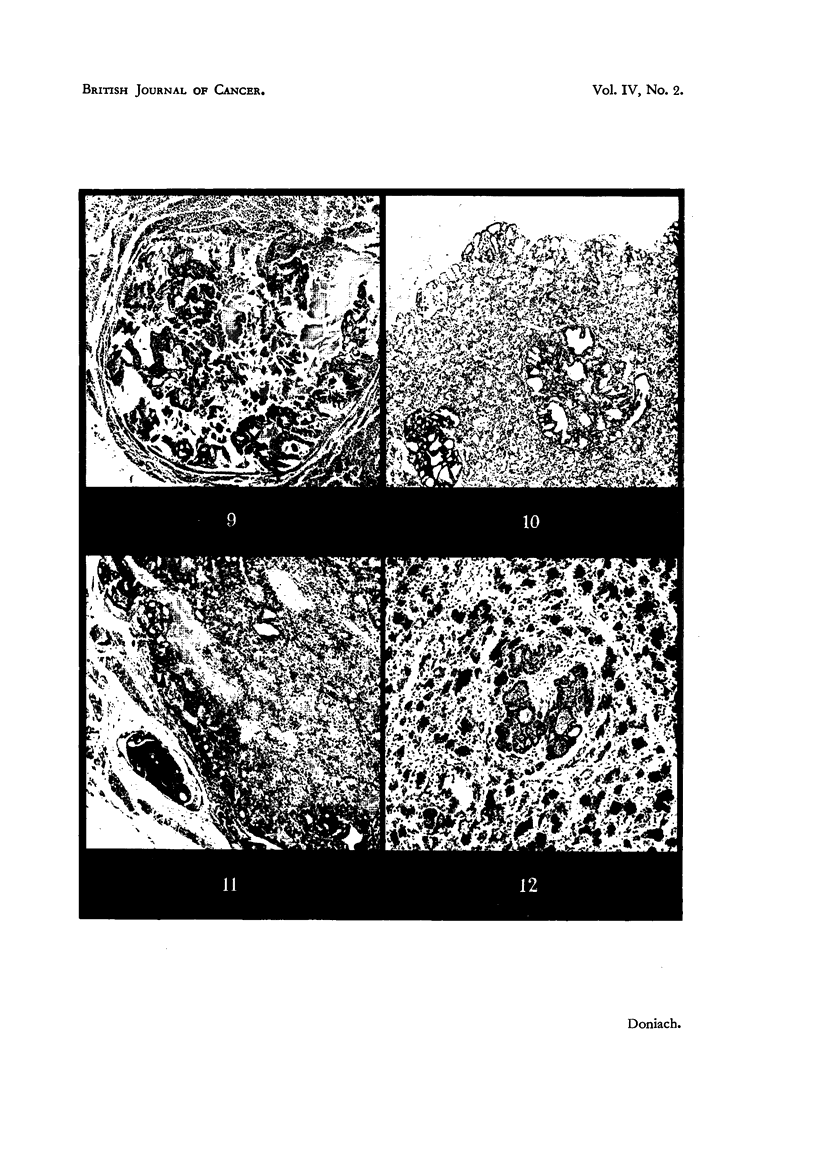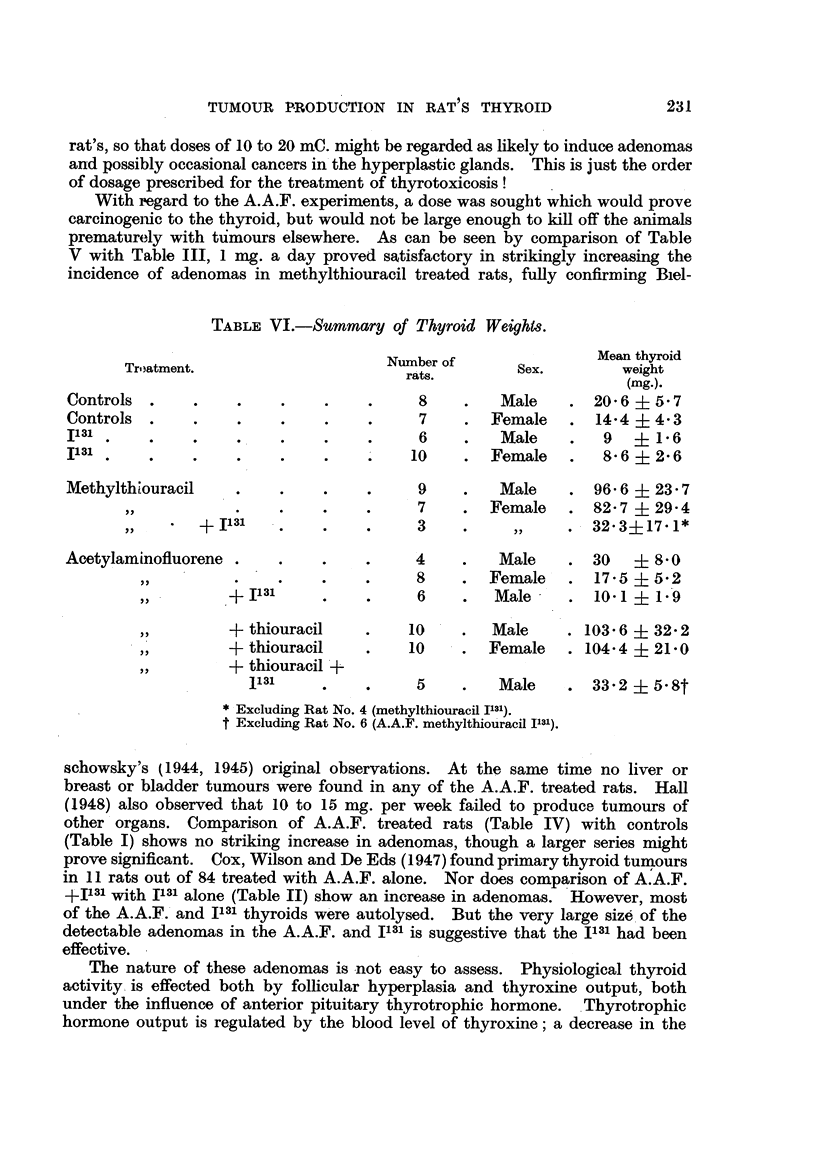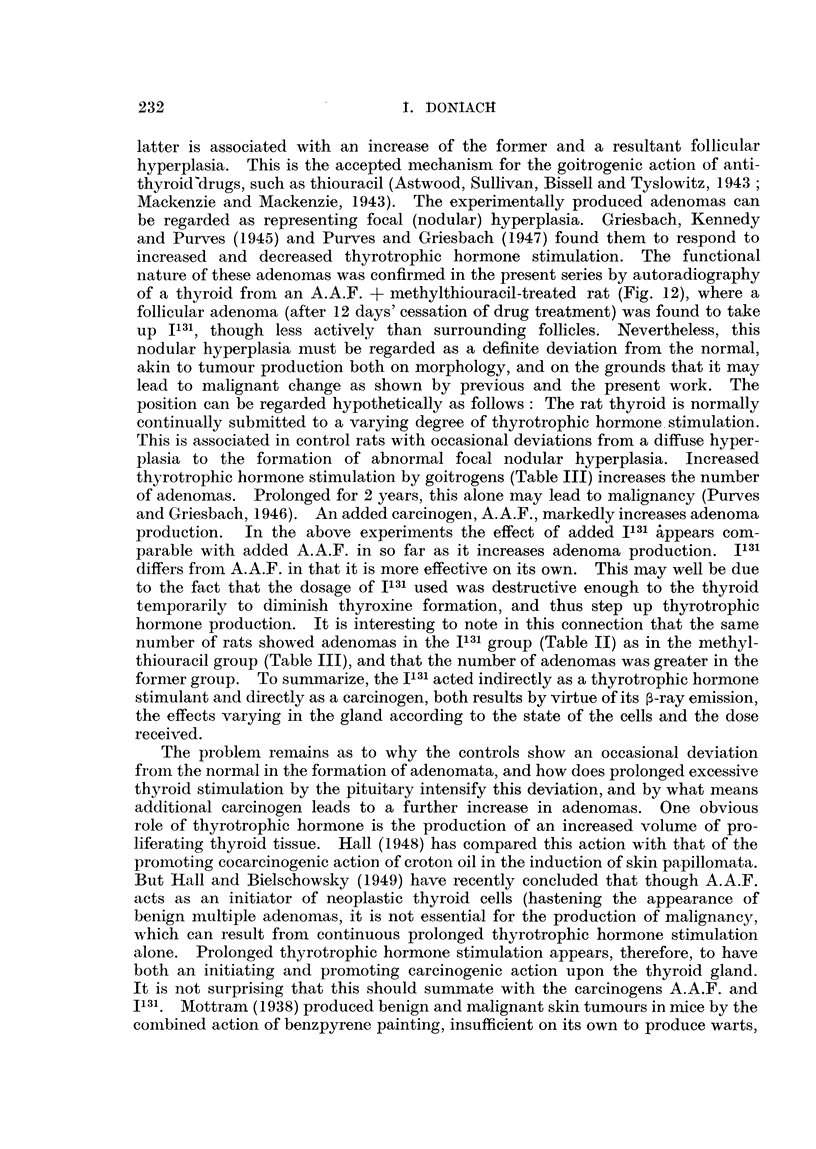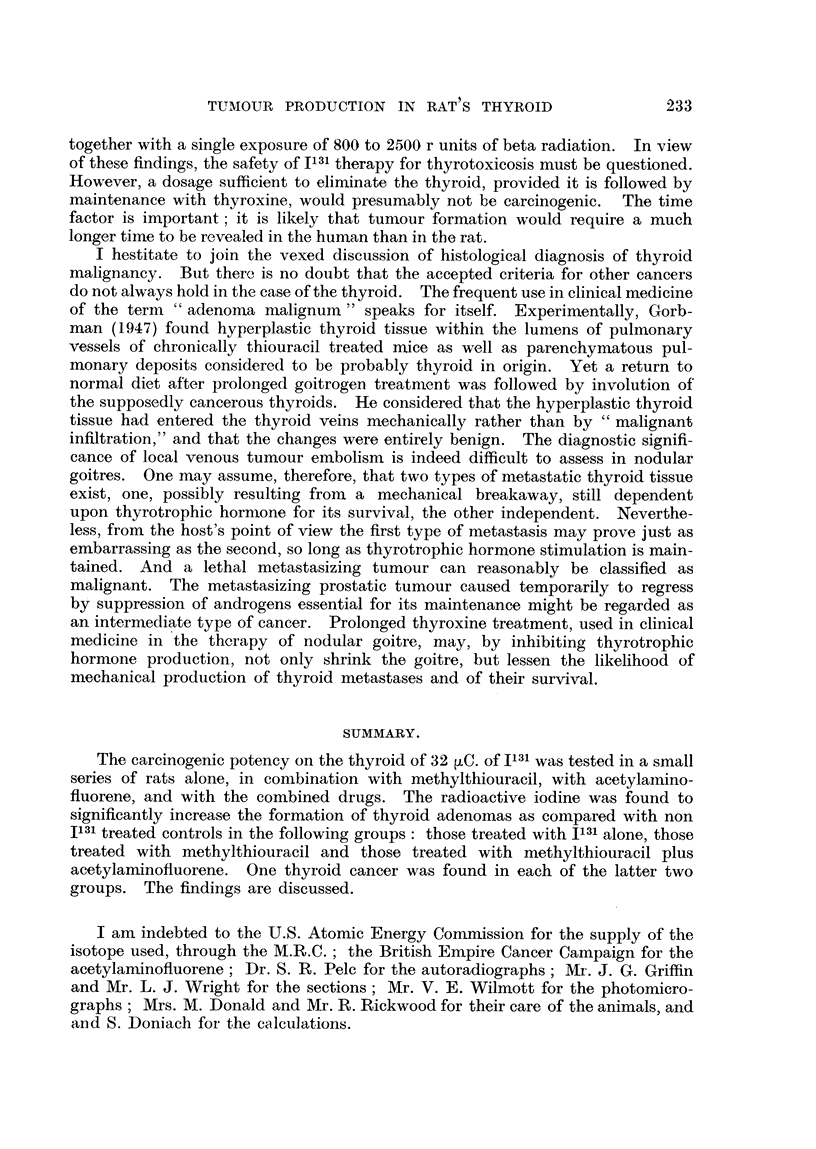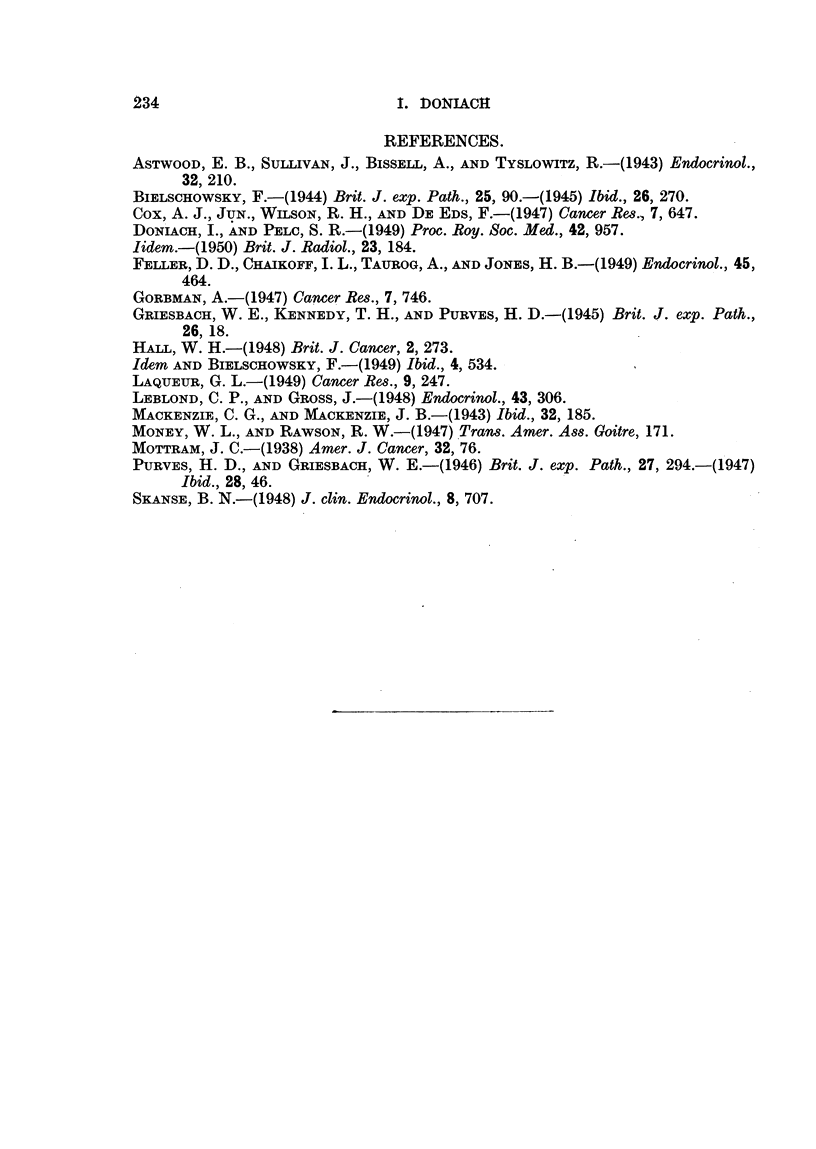# The Effect of Radioactive Iodine Alone and in Combination with Methylthiouracil and Acetylaminofluorene upon Tumour Production in the Rat's Thyroid Gland

**DOI:** 10.1038/bjc.1950.22

**Published:** 1950-06

**Authors:** I. Doniach

## Abstract

**Images:**


### THE EFFECT OF RADIOACTIVE IODINE ALONE AND IN COMBINATION WITH METHYLTHIOURACIL AND ACETYLAMINOFLUORENE UPON TUMOUR PRODUCTION IN THE RAT'S THYROID GLAND.

I. DONIACH.

*From the Pathology Department, Post-Graduate Medical School of London, W.12.*

Received for publication May 6, 1950.

THE object of the following experiments was an attempt to determine whether radioactive iodine, $I^{131}$, is carcinogenic to the rat's thyroid gland. It seemed likely that $I^{131}$ would prove so since it is specifically concentrated in the thyroid, whose cells are thereby submitted to a course of β-ray irradiation. The problem is important, since we do not yet know the carcinogenic hazard of $I^{131}$ in clinical medicine, where it is being increasingly used in both tracer and therapeutic doses. Hyperplasia and adenomas of the thyroid have been produced by goitrogens (Griesbach, Kennedy and Purves, 1945), and by goitrogens together with the carcinogen acetylaminofluorene (A.A.F.) (Bielschowsky, 1944). Both treatments have produced malignant tumours (Purves and Griesbach, 1946), the addition of A.A.F. enhancing this effect (Bielschowsky, 1945). Part of the mechanism of tumour production in those experiments was the thyroid hyperplasia induced by the goitrogens. When the following work was started in February, 1948, there were discrepancies in the literature as to the incidence of thyroid tumours in untreated rats and the carcinogenic potencies of goitrogens, which were due to the different rat strains, ages and living conditions, times of application of the drugs and criteria of malignancy used. In view of the discrepancies and in order to compare the action of $I^{131}$ with A.A.F. on normal and hyperplastic thyroids, the rats were divided into 8 groups, treated as follows:

(1) Controls. (2) $I^{131}$. (3) Methylthiouracil. (4) Methylthiouracil + $I^{131}$. (5) A.A.F. (6) A.A.F. + $I^{131}$. (7) A.A.F. + methylthiouracil. (8) A.A.F. + methylthiouracil + $I^{131}$.

### MATERIAL AND METHODS.

A total of 113 hooded rats of the Lister strain were used, pen-inbred from one male and two females. They were fed on "Research (Rat) Cubes" from Heygate & Sons, Northampton, with additional greens. The 2-methyl-4-thiouracil was given as a saturated solution in the drinking tap-water. The 2-acetylaminofluorene was given as a suspension, 1 mg. per 10 ml. of a 0·75 per cent gum-acacia solution in the drinking tap-water. The suspension was made by adding an acetone solution of the aminofluorene to the gum-acacia solution in a beaker and allowing the acetone to evaporate off overnight. The combined drugs were given as a saturated solution of methylthiouracil in the aminofluorene suspension. The rats drank about 8 to 12 ml. fluids per day. The radioactive iodine $I^{131}$ was carrier-free and given in a dosage of 16 μC., by intraperitoneal injection in 1 ml. of water. This dose is 10 μC. as received from Oak Ridge in 1948, which must be regarded as 16 μC. in accordance with an announcement from Oak Ridge in Spring, 1949.

The experiment was started with 20 rats, which were added to during the following 4 months. The animals were arranged in the groups as described, subdivided into sexes. Halfway through the experiments they were given a six weeks, " summer holiday " off all drugs. The radioactive iodine was injected 48 hours before starting the thiouracil, to allow iodide accumulation into the thyroid gland. The $I^{131}$ injection was repeated after the " summer holiday," so that the total dosage of radioactive iodine was 32 μC. per rat, given in 2 doses of 16 μC. separated by a 5½ months' interval. The rats were killed by coal gas at an average age of 15 months, having had treatment for an average of 13 months (including the rest period). All animals, whether they had died or been killed, were autopsied. The trachea and thyroid attached was fixed in Helly's fluid ; the thyroid was then dissected off the trachea and weighed to the nearest milligram. The thyroids were embedded in wax, serially cut at 5μ and mounted on to slides as ribbons of 8 to 14 sections, including both lobes. Every fourth slide was stained by haemalum and eosin. Sections were also taken from the lungs, of a number of pituitaries and of all macroscopically abnormal organs. The number of rats used was 98 to obtain data for the main experiment, at the end of which the remaining 15 from various groups were put aside for study of the thyroid $I^{131}$ uptake by direct measurement and autoradiography. The autoradiographs were made by the stripping film technique described in detail by Doniach and Pelc (1950), the rats being injected with 16 μC. carrier-free $I^{131}$ 24 hours before being killed.

### RESULTS.

The indication of adenomas by plus signs in Tables I, II, III, IV and V is not devised to be any more than a crude indication of tumour incidence ; the object was to seek for major rather than minor differences resulting from the various treatments of these small numbers of rats. I have not given a detailed description of the histology and histogenesis of the adenomas, because in addition to the references cited in the introduction, there have appeared further detailed illustrated descriptions by Purves and Griesbach (1947), Money and Rawson (1947), Laqueur (1949), and Hall and Bielschowsky (1949). Autopsies on the majority of the animals found dead revealed no more than wasting and post-mortem autolysis ; pathological findings are recorded in the text.

### *Controls* (*Table I*).

The thyroid sections in general showed large peripheral follicles, rich in deeply eosinophilic colloid, lined by a cuboidal epithelium and smaller central follicles containing a paler, finely vacuolated colloid lined by a slightly higher cuboidal epithelium. In each of 4 rats a sharply demarcated single or sometimes double spherical focus was found 50 to 100 μ across of closely packed spheroidal cells with voluminous eosinophilic cytoplasm and vesicular nuclei, showing an occasional mitosis (Fig. 1). These were seen to be solid on serial section and surrounded by a delicate reticulin fibre capsule on silver stain. They appeared to have arisen by dedifferentiation and proliferation of the lining cells of one or two contiguous follicles and were regarded as solid adenomas. One adenoma, Female Rat 3, showed focal differentiation of newly-formed microfollicles. Solid adenomas were found more frequently in the treated rats, where some showed a tendency to a trabecular arrangement of their constituent cells.

TABLE I.—*Controls.*

| Rat. | Sex. | Age (months). | Mode of death. | Body weight (g.). | Thyroid weight (mg.). | Thyroid adenomas. |
|---|---|---|---|---|---|---|
| 1 | Male | 12 | Died | 135 | 10 | *Nil* |
| 2 | ,, | 13 | Killed | 305 | 20 | ,, |
| 3 | ,, | 13 | ,, | 305 | 19 | ,, |
| 4 | ,, | 13 | ,, | 300 | 19 | ,, |
| 5 | ,, | 13 | ,, | 300 | 24 | ,, |
| 6 | ,, | 13 | ,, | 340 | 21 | + |
| 7 | ,, | 13 | ,, | 295 | 20 | + |
| 8 | ,, | 17 | ,, | 360 | 32 | *Nil* |
| 1 | Female | 14 | ,, | 180 | 12 | ,, |
| 2 | ,, | 14 | ,, | 210 | 13 | ,, |
| 3 | ,, | 14 | ,, | 200 | 12 | + |
| 4 | ,, | 14 | ,, | 170 | 10 | *Nil* |
| 5 | ,, | 14 | ,, | 200 | 12 | ,, |
| 6 | ,, | 17 | ,, | 255 | 19 | + |
| 7 | ,, | 17 | ,, | 250 | 23 | *Nil* |

+ Represents the finding of 1 adenoma per gland.

*Radioactive Iodine* (*Table II*).

The body weights were normal, but the thyroid glands were only half the weights of the controls as shown in Table VI. The thyroid tissue in general differed from the controls in showing a greater variation in cell height, follicle size and colloid storage. Adenomas were present in at least 10 out of the 16 rats. They were mostly follicular in type, consisting of foci of colloid containing

TABLE II.—*Radioactive Iodine.*

| Rat. | Sex. | Age (months). | Treatment* (months). | Mode of death. | Body weight (g.). | Thyroid weight. (mg.). | Thyroid adenomas. |
|---|---|---|---|---|---|---|---|
| 1 | Male | 14 | 12 | Died | 250 | 9 | *Nil* |
| 2 | ,, | 15 | 13 | Killed | 370 | 7 | ++ |
| 3 | ,, | 15 | 13 | ,, | 365 | 8 | +++ |
| 4 | ,, | 16 | 14 | ,, | 350 | 11 | ++ |
| 5 | ,, | 16 | 14 | ,, | 340 | 8 | +++ |
| 6 | ,, | 16 | 14 | ,, | 335 | 11 | +++ |
| 1 | Female | 16½ | 14 | ,, | 230 | 10 | *Nil* |
| 2 | ,, | 16½ | 14 | ,, | 255 | 12 | ,, |
| 3 | ,, | 16½ | 14 | ,, | 230 | 11 | ,, |
| 4 | ,, | 16½ | 14 | ,, | 250 | 7 | ++ |
| 5 | ,, | 16½ | 14 | ,, | 240 | 6 | *Nil* |
| 6 | ,, | 16½ | 14 | ,, | 250 | 5 | + |
| 7 | ,, | 17 | 14½ | ,, | 120 | 5 | + |
| 8 | ,, | 17 | 14½ | ,, | 240 | 10 | +++ |
| 9 | ,, | 17 | 14½ | ,, | 240 | 7 | +++ |
| 10 | ,, | 18 | 15½ | ,, | 220 | 13 | *Nil* |

* Number of months before the rats were killed when they received the first of their two injections of radioactive iodine.

+ Represents 1 adenoma per gland; ++, 2 or 3 adenomas; +++, multiple adenomas.

follicles and tubules lined by closely packed cuboidal and low columnar cells with small darkly staining nuclei and basophilic cytoplasm (Fig. 2). In many areas it was difficult to differentiate the adenomas, and the number of plus signs awarded in the thyroid adenoma column of Table II is probably an underestimate. The pre-adenomatous foci (Fig. 2) were similar to those described by Laqueur (1949), consisting of a few follicles larger than their neighbours lined by closely packed cells containing hyperchromatic nuclei. Female Rat No. 5 showed a parathyroid adenoma; her bones were not sectioned. At autopsy Male Rat No. 1 showed cirrhosis of the liver and a malignant lymphoma massively involving both the abdominal and thoracic lymph-nodes.

*Methylthiouracil (Table III).*

The rats were lively and appeared healthy, but they only developed to half the body weight of the controls. Their thyroid glands (Table VI) were five times heavier than those of the controls. Sections showed a remarkable follicular hyperplasia, gross vascularity and almost total colloid depletion. The follicular epithelium was columnar and higher in the central follicles, whose lumens were thereby smaller than those of the peripheral follicles. Most glands showed focal fibrous thickening of the capsule, which often traversed the parenchyma and enclosed thyroid follicles, giving rise to a spurious appearance of infiltration by parenchymatous tissue and of adenoma formation (Fig. 3). In addition, the very close proximity of some follicles to large thin-walled subcapsular venous

TABLE III.—*Methylthiouracil.*

| Rat. | Sex. | Age (months). | Treatment (months). | Mode of death | Body weight (g.). | Thyroid weight. (mg.). | Thyroid adenomas. |
|---|---|---|---|---|---|---|---|
| 1 | Male | 11 | 9 | Died | 120 | 46 | *Nil* |
| 2 | „ | 12 | 11 | Killed | 130 | 72 | + |
| 3 | „ | 13 | 12 | „ | 170 | 114 | *Nil* |
| 4 | „ | 13 | 12 | „ | 160 | 127 | ++ |
| 5 | „ | 13 | 12 | „ | 170 | 114 | *Nil* |
| 6 | „ | 13 | 11½ | Died | 105 | 85 | „ |
| 7 | „ | 16 | 14 | Killed | 170 | 107 | ++ |
| 8 | „ | 16 | 14 | „ | 160 | 106 | *Nil* |
| 9 | „ | 16 | 14 | „ | 160 | 99 | + |
| 1 | Female | 12 | 10 | „ | 100 | 29 | *Nil* |
| 2 | „ | 16 | 15 | „ | 155 | 100 | + |
| 3 | „ | 16 | 15 | „ | 125 | 120 | + |
| 4 | „ | 16 | 15 | „ | 135 | 99 | + |
| 5 | „ | 16 | 15 | „ | 85 | 53 | ++ |
| 6 | „ | 16 | 15 | „ | 115 | 76 | + |
| 7 | „ | 16 | 15 | „ | 125 | 102 | ++ |
| *Methylthiouracil + Radioactive Iodine.* | | | | | | | |
| 1 | Female | 14½ | 12 | Died | 150 | .. | Thyroid lost |
| 2 | „ | 16½ | 14 | Killed | 135 | 45 | +++ |
| 3 | „ | 16½ | 14 | „ | 125 | 44 | +++ |
| 4 | „ | 16½ | 14 | „ | 120 | 364 | ++++ cancer |
| 5 | „ | 16½ | 14 | „ | 115 | 8 | +++ |

+, ++, +++ as in Table II.
++++ Represents adenomatous replacement of most of the thyroid gland.

sinusoids mimicked a pre-invasive change (Fig. 3). However, the cells of these extra and intracapsular and juxta-venous follicles were quite innocent in appearance, and in no way different from those constituting the main mass of the glands. Many arteries showed a remarkable muscular hyperplasia of their walls and reduction of their lumens. The pericapsular tissue of a few glands was inflamed and infiltrated with polymorphs. Solid and follicular adenomas, mostly the latter, were present in 10 of the 16 glands. Some of the adenomatous follicles were grossly distended; a few contained eosinophilic colloid. Many of the adenomas were grouped around a large blood vessel (Fig. 4). At autopsy Male Rat No. 6 showed massive suppuration of the peri-urethral glands.

*Methylthiouracil and radioactive iodine* (*Table III*).

The body weights of these 5 rats did not differ significantly from those treated with methylthiouracil alone. The thyroid glands were smaller except for the cancerous one, though larger than those of untreated controls (Table VI). The thyroid of Rat No. 1, which was lost, was noted at autopsy to be only moderately enlarged. The thyroids of Rats 2, 3 and 5 showed a typical thiouracil follicular hyperplasia and colloid depletion of surviving thyroid tissue. Most of the tissue, however, was replaced by very numerous adenomas, solid, mixed solid and follicular, follicular, and papilliferous cystic (Fig. 5). Many were rich in eosinophilic colloid. There were areas present of an appearance suggestive of pre- or early adenomatous change, difficult to differentiate from adenomas proper. The effect of the $I^{131}$ was such that every one of the glands in this admittedly small group showed a most striking increase in adenoma formation as compared with the rats treated with methylthiouracil alone. Both lobes of the enormously enlarged thyroid of Rat No. 4 were almost entirely replaced by a fantastic mixture of all varieties of large adenomas, some of them very cellular. Many veins outside the gland were plugged with mixed solid and microfollicular tumour (Fig. 6). Growth of the solid adenoma type was present in random pulmonary arteries (Fig. 7), and one deposit of similar tumour had enlarged and mostly replaced one of the adrenal glands. This rat was regarded as having developed a cancer of the thyroid.

*Acetylaminofluorene* (*Table IV*).

The body weights were within normal limits. The thyroid weights varied, as did the associated histology. Most of them appeared similar to controls, but the thyroids of Male Rat No. 6 and Female Rats Nos. 6, 7 and 8 showed a definite moderate hyperplasia and colloid depletion. Adenomas were present in 7 of the 14 rats, a few were follicular colloid containing (Fig. 8), most were solid with early follicle formation. It was difficult either to diagnose or rule out the presence of adenomas in the somewhat autolysed thyroids of the 4 rats which had died.

*Acetylaminofluorene and radioactive iodine* (*Table IV*).

Of the 6 rats 5 were wasted and died. Rats 3 and 5 showed massive suppuration of the peri-urethral glands. Autolysis rendered the histological diagnosis of adenomas difficult. They were mostly large colloid secreting, follicular in type (Fig. 9).

TABLE IV.—*Acetylaminofluorene.*

| Rat. | Sex. | Age (months). | Treatment (months). | Mode of death. | Body weight (g.). | Thyroid weight (mg.). | Thyroid adenomas. |
|---|---|---|---|---|---|---|---|
| 1 | Male | 6½ | 4 | Died | 110 | .. | *Nil* |
| 2 | ,, | 8 | 7 | ,, | 110 | .. | ,, |
| 3 | ,, | 10 | 9 | ,, | 170 | 17 | ,, |
| 4 | ,, | 15 | 14 | Killed | 300 | 26 | + |
| 5 | ,, | 15 | 14 | ,, | 270 | 26 | *Nil* |
| 6 | ,, | 17½ | 14½ | ,, | 360 | 44 | + |
| 1 | Female | 12 | 11 | ,, | 145 | 10 | ++ |
| 2 | ,, | 15 | 14 | Died | 120 | 13 | *Nil* |
| 3 | ,, | 15 | 14 | Killed | 185 | 16 | ,, |
| 4 | ,, | 15 | 14 | ,, | 180 | 15 | + |
| 5 | ,, | 15 | 14 | ,, | 195 | 15 | + |
| 6 | ,, | 17 | 15 | ,, | 250 | 26 | ++ |
| 7 | ,, | 17 | 15 | ,, | 250 | 24 | ++ |
| 8 | ,, | 17 | 15 | ,, | 225 | 21 | *Nil* |
| *Acetylaminofluorene + Radioactive Iodine.* | | | | | | | |
| 1 | Male | 12 | 9½ | Died | 160 | 9 | *Nil* |
| 2 | ,, | 15 | 12½ | ,, | 190 | 12 | ++ |
| 3 | ,, | 14½ | 12 | ,, | 250 | 12 | *Nil* |
| 4 | ,, | 15½ | 13 | ,, | 200 | 12 | ++ |
| 5 | ,, | 16 | 13½ | ,, | 180 | 8 | *Nil* |
| 6 | ,, | 16½ | 14 | Killed | 300 | 8 | ++ |

+, ++ as in Table II.

*Acetylaminofluorene and methylthiouracil* (*Table V*).

The body weights and thyroid weights were comparable to those of rats treated with methylthiouracil alone. The general thyroid histology also closely resembled that of the methylthiouracil treated rats showing hyperplasia, colloid depletion, thickened capsules and vessels. Pre-adenomatous hyperplasia was a little more marked. The thyroids of all the rats treated for 10 months or more abounded in multiple medium-sized adenomas, solid, follicular and mixed (Fig. 10). Female Rats 1 and 2 died of widespread involvement of thoracic and abdominal lymph-nodes by malignant lymphoma. Male Rat 1 showed kidney suppuration and Male Rat 2 suppuration of the peri-urethral glands.

*Acetylaminofluorene, methylthiouracil and radioactive iodine* (*Table V*).

The thyroid weights (excluding Rat No. 6) were a third of those of the previous group, but still three times heavier than those of rats treated with $I^{131}$ alone (Table VI). All contained multiple large adenomas, which in Rats 2, 3, 4, 5 and 6 had almost totally replaced normal thyroid tissue. They were similar in appearance to those seen in the rats treated with methylthiouracil and $I^{131}$. Rat No. 6, whose thyroid was three times heavier than the rest of the group, showed plugs of tumour cells of the solid adenoma type within the lumen of veins outside the thyroid gland (Fig. 11). Random lung sections were free of growth. This rat was regarded as having developed a cancer of the thyroid. Rat No. 3 died with massive suppuration of the peri-urethral glands.

The results of the studies on the 15 supplementary rats will not be detailed in this paper, except that one autoradiograph, considered relevant to the discussion, is included in the illustrations (Fig. 12).

TABLE V.—*Acetylaminofluorene + Methylthiouracil.*

| Rat. | Sex. | Age (months). | Treatment (months). | Mode of death. | Body weight (g.). | Thyroid weight (mg.). | Thyroid adenomas. |
|---|---|---|---|---|---|---|---|
| 1 | Male | 9 | 7½ | Died | 110 | 100 | *Nil* |
| 2 | ,, | 10½ | 8 | ,, | 120 | 60 | ,, |
| 3 | ,, | 14 | 11½ | Killed | 150 | 83 | +++ |
| 4 | ,, | 14½ | 12 | Died | 130 | 67 | ++ |
| 5 | ,, | 14½ | 13 | Killed | 165 | 77 | +++ |
| 6 | ,, | 14½ | 13 | ,, | 185 | 168 | +++ |
| 7 | ,, | 14½ | 13 | ,, | 165 | 100 | +++ |
| 8 | ,, | 15 | 12½ | ,, | 200 | 129 | +++ |
| 9 | ,, | 15 | 12½ | ,, | 180 | 136 | +++ |
| 10 | ,, | 17½ | 15 | ,, | 150 | 116 | +++ |
| 1 | Female | 11½ | 10 | Died | 120 | 60 | +++ |
| 2 | ,, | 11½ | 10 | ,, | .. | 117 | +++ |
| 3 | ,, | 14½ | 13 | Killed | 160 | 119 | +++ |
| 4 | ,, | 14½ | 13 | ,, | 125 | 125 | ++++ |
| 5 | ,, | 14½ | 13 | ,, | 130 | 120 | +++ |
| 6 | ,, | 15 | 13 | Died | 110 | 108 | +++ |
| 7 | ,, | 15 | 13 | Killed | 125 | 124 | +++ |
| 8 | ,, | 15 | 13 | ,, | 130 | 85 | +++ |
| 9 | ,, | 15 | 13 | ,, | 155 | 107 | ++++ |
| 10 | ,, | 15½ | 11½ | Died | 110 | 79 | +++ |
| *Acetylaminofluorene + Methylthiouracil + Radioactive Iodine.* | | | | | | | |
| 1 | Male | 14 | 12 | Died | 120 | 40 | +++ |
| 2 | ,, | 14½ | 11½ | ,, | 130 | 36 | ++++ |
| 3 | ,, | 14½ | 12 | ,, | 125 | 27 | ++++ |
| 4 | ,, | 14½ | 12 | Killed | 140 | 36 | ++++ |
| 5 | ,, | 15 | 13 | Died | 130 | 27 | ++++ |
| 6 | ,, | 16 | 14 | Killed | 175 | 99 | ++++ cancer |

+, ++, +++, ++++ as in Tables II and III.

DISCUSSION.

The above results show that radioactive iodine increased the incidence of thyroid adenomas in all groups except the A.A.F., which will be discussed later. The adenomas were larger in size as well as increased in number, and showed evidence of malignancy by a gross increase in size and by dissemination outside the thyroid in two instances. The assessment of radiation dosage administered to the thyroid by $I^{131}$ is a difficult one. In the first place the percentage uptake varies with the iodine content of the diet and the surrounding temperature. We have found iodine uptake by the thyroid 24 hours after administering $I^{131}$ varying from 10 per cent in hot weather to 30 per cent when it was cool. Secondly, as shown by autoradiography (Leblond and Gross, 1948 ; Doniach and Pelc, 1949), there is a wide variation in concentration from follicle to follicle ; the peripheral follicles in the rat take up considerably less iodine than the central ones. This is only partly balanced by the more rapid disappearance of iodine from the central follicles. The radiation effects are due mostly to $\beta$ radiation, since most of the $\gamma$ rays, which are much more penetrating, will not be absorbed in the small thyroid of the rat. This uneven distribution of $I^{131}$ almost certainly accounts for the remarkable resistance of the thyroid to radiation as computed in roentgen equivalents. The associated variation in cell activity may similarly account for the comparative resistance to X-irradiation. Destruction of active cells is presumably followed by a regenerative activity of surviving resting cells, and the gland will function normally. Feller, Chaikoff, Taurog and Jones (1949) have recently estimated that after injection of 30 $\mu$C. of $I^{131}$ the rat thyroid receives an overall dose of 28,000 reps (roentgen equivalents physical) during the ensuing 10 days. They found that this radiation did not interfere at all with the ability of the thyroids to take up further iodine, produced no histological changes, and showed no alteration of thyroid function as gauged by various physiological tests. Their rats averaged 40 per cent maximal uptake of $I^{131}$, ours was nearer 20 per cent, and our total $I^{131}$ injected was 32, theirs was 30 $\mu$C., therefore our dosage was roughly 15,000 roentgen equivalents physical. Skanse (1948) tested the effect on the chick thyroid of injections of 1, 10 and 50 $\mu$C. of $I^{131}$. He calculated that the total doses received by the glands were respectively 1700, 13,000 and 60,000 reps. In these young growing animals the 10 $\mu$C. $I^{131}$ (13,000 reps) produced a significant inhibition of thyroid gland growth, though not of function, after 16 days. From the long-term view in our experiments this dosage proved damaging, as shown in Table VI, where it can be seen that the thyroid weights of all $I^{131}$ treated rats in all groups was one-half to one-third of their respective controls. Clearly though, enough thyroid had survived to maintain normal growth of the rats, since the body-weights did not differ from their respective controls in spite of the fact that the first dose of $I^{131}$ was administered at an average age of $2\frac{1}{2}$ months. The human thyroid is 1000 times heavier than the

### EXPLANATION OF PLATES.

FIG. 1.—Thyroid of Female Control Rat No. 6 showing a " solid " cellular adenoma. × 145.

FIG. 2.—Thyroid of Male $I^{131}$ Rat No. 3 showing a trabecular adenoma below and to the right, a follicular adenoma above and to the left and a parathyroid above and to the right. Between the adenomas are a few pre-adenomatous foci consisting of two or three large follicles lined by closely packed hyperchromatic cells. × 36.

FIG. 3.—Thyroid of male methylthiouracil Rat No. 8 showing a thickened capsule separating groups of follicles from the main gland. The follicles in general are small, hyperplastic and empty of colloid. They are closely apposed to the endothelium of a large subcapsular venous sinusoid in the upper left half of the photomicrograph. One artery, traversing the gland, shows a marked muscular hyperplasia of its wall. Above it lies an elongated parathyroid embedded in the gland. × 40.

FIG. 4.—Thyroid of Male methylthiouracil Rat No. 9 showing follicular hyperplasia, thickening of the capsule and adenoma formation round a large blood vessel. × 38.

FIG. 5.—Thyroid of Female methylthiouracil $I^{131}$ Rat No. 2 showing multiple adenomas of varying morphology in a hyperplastic gland. × 40.

FIG. 6.—Thyroid of Female methylthiouracil $I^{131}$ Rat No. 4 showing adenomatous replacement of the gland, a thickened capsule and a plug of tumour tissue filling a pericapsular vein, above and to the right. × 40.

FIG. 7.—Lung of Female methylthiouracil $I^{131}$ Rat No. 4 showing emboli of tumour cells in branches of the pulmonary artery. × 40.

FIG. 8.—Thyroid of Male A.A.F. Rat No. 4 showing a colloid containing follicular adenoma in an otherwise normal gland. × 45.

FIG. 9.—Thyroid of Male A.A.F. $I^{131}$ Rat No. 2 showing a large papillary colloid secreting adenoma in an autolysed gland. × 40.

FIG. 10.—Thyroid of Female A.A.F. methylthiouracil Rat No. 3 showing multiple adenomas in a hyperplastic gland with a thickened capsule. × 40.

FIG. 11.—Thyroid of Male A.A.F. methylthiouracil $I^{131}$ Rat No. 6 showing adenomatous replacement of the gland and a plug of tumour tissue in a neighbouring vein, below and to the left. × 40.

FIG. 12.—Haemalum stained autoradiograph of a 15-month-old female A.A.F. methylthiouracil rat killed 12 days after cessation of 13 months' treatment, showing intense blackening over the colloid of the normal follicles and less intense but definite blackening over the colloid secreted by the adenoma lying in the centre of the photomicrograph. The rat had received 16 $\mu$C. of $I^{131}$ 24 hours before being killed. × 40.

Doniach.

5

6

7

8

9

10

11

12

rat's, so that doses of 10 to 20 mC. might be regarded as likely to induce adenomas and possibly occasional cancers in the hyperplastic glands. This is just the order of dosage prescribed for the treatment of thyrotoxicosis!

With regard to the A.A.F. experiments, a dose was sought which would prove carcinogenic to the thyroid, but would not be large enough to kill off the animals prematurely with tumours elsewhere. As can be seen by comparison of Table V with Table III, 1 mg. a day proved satisfactory in strikingly increasing the incidence of adenomas in methylthiouracil treated rats, fully confirming Bielschowsky's (1944, 1945) original observations. At the same time no liver or breast or bladder tumours were found in any of the A.A.F. treated rats. Hall (1948) also observed that 10 to 15 mg. per week failed to produce tumours of other organs. Comparison of A.A.F. treated rats (Table IV) with controls (Table I) shows no striking increase in adenomas, though a larger series might prove significant. Cox, Wilson and De Eds (1947) found primary thyroid tumours in 11 rats out of 84 treated with A.A.F. alone. Nor does comparison of A.A.F. $+I^{131}$ with $I^{131}$ alone (Table II) show an increase in adenomas. However, most of the A.A.F. and $I^{131}$ thyroids were autolysed. But the very large size of the detectable adenomas in the A.A.F. and $I^{131}$ is suggestive that the $I^{131}$ had been effective.

TABLE VI.—*Summary of Thyroid Weights.*

| Treatment. | Number of rats. | Sex. | Mean thyroid weight (mg.). |
|---|---|---|---|
| Controls | 8 | Male | $20\cdot6 \pm 5\cdot7$ |
| Controls | 7 | Female | $14\cdot4 \pm 4\cdot3$ |
| $I^{131}$ | 6 | Male | $9 \pm 1\cdot6$ |
| $I^{131}$ | 10 | Female | $8\cdot6 \pm 2\cdot6$ |
| Methylthiouracil | 9 | Male | $96\cdot6 \pm 23\cdot7$ |
| ,, | 7 | Female | $82\cdot7 \pm 29\cdot4$ |
| ,, $+ I^{131}$ | 3 | ,, | $32\cdot3 \pm 17\cdot1$* |
| Acetylaminofluorene | 4 | Male | $30 \pm 8\cdot0$ |
| ,, | 8 | Female | $17\cdot5 \pm 5\cdot2$ |
| ,, $+ I^{131}$ | 6 | Male | $10\cdot1 \pm 1\cdot9$ |
| ,, + thiouracil | 10 | Male | $103\cdot6 \pm 32\cdot2$ |
| ,, + thiouracil | 10 | Female | $104\cdot4 \pm 21\cdot0$ |
| ,, + thiouracil + $I^{131}$ | 5 | Male | $33\cdot2 \pm 5\cdot8$† |

* Excluding Rat No. 4 (methylthiouracil $I^{131}$).
† Excluding Rat No. 6 (A.A.F. methylthiouracil $I^{131}$).

The nature of these adenomas is not easy to assess. Physiological thyroid activity is effected both by follicular hyperplasia and thyroxine output, both under the influence of anterior pituitary thyrotrophic hormone. Thyrotrophic hormone output is regulated by the blood level of thyroxine; a decrease in the latter is associated with an increase of the former and a resultant follicular hyperplasia. This is the accepted mechanism for the goitrogenic action of antithyroid drugs, such as thiouracil (Astwood, Sullivan, Bissell and Tyslowitz, 1943 ; Mackenzie and Mackenzie, 1943). The experimentally produced adenomas can be regarded as representing focal (nodular) hyperplasia. Griesbach, Kennedy and Purves (1945) and Purves and Griesbach (1947) found them to respond to increased and decreased thyrotrophic hormone stimulation. The functional nature of these adenomas was confirmed in the present series by autoradiography of a thyroid from an A.A.F. + methylthiouracil-treated rat (Fig. 12), where a follicular adenoma (after 12 days' cessation of drug treatment) was found to take up $I^{131}$, though less actively than surrounding follicles. Nevertheless, this nodular hyperplasia must be regarded as a definite deviation from the normal, akin to tumour production both on morphology, and on the grounds that it may lead to malignant change as shown by previous and the present work. The position can be regarded hypothetically as follows : The rat thyroid is normally continually submitted to a varying degree of thyrotrophic hormone stimulation. This is associated in control rats with occasional deviations from a diffuse hyperplasia to the formation of abnormal focal nodular hyperplasia. Increased thyrotrophic hormone stimulation by goitrogens (Table III) increases the number of adenomas. Prolonged for 2 years, this alone may lead to malignancy (Purves and Griesbach, 1946). An added carcinogen, A.A.F., markedly increases adenoma production. In the above experiments the effect of added $I^{131}$ appears comparable with added A.A.F. in so far as it increases adenoma production. $I^{131}$ differs from A.A.F. in that it is more effective on its own. This may well be due to the fact that the dosage of $I^{131}$ used was destructive enough to the thyroid temporarily to diminish thyroxine formation, and thus step up thyrotrophic hormone production. It is interesting to note in this connection that the same number of rats showed adenomas in the $I^{131}$ group (Table II) as in the methylthiouracil group (Table III), and that the number of adenomas was greater in the former group. To summarize, the $I^{131}$ acted indirectly as a thyrotrophic hormone stimulant and directly as a carcinogen, both results by virtue of its $\beta$-ray emission, the effects varying in the gland according to the state of the cells and the dose received.

The problem remains as to why the controls show an occasional deviation from the normal in the formation of adenomata, and how does prolonged excessive thyroid stimulation by the pituitary intensify this deviation, and by what means additional carcinogen leads to a further increase in adenomas. One obvious role of thyrotrophic hormone is the production of an increased volume of proliferating thyroid tissue. Hall (1948) has compared this action with that of the promoting cocarcinogenic action of croton oil in the induction of skin papillomata. But Hall and Bielschowsky (1949) have recently concluded that though A.A.F. acts as an initiator of neoplastic thyroid cells (hastening the appearance of benign multiple adenomas, it is not essential for the production of malignancy, which can result from continuous prolonged thyrotrophic hormone stimulation alone. Prolonged thyrotrophic hormone stimulation appears, therefore, to have both an initiating and promoting carcinogenic action upon the thyroid gland. It is not surprising that this should summate with the carcinogens A.A.F. and $I^{131}$. Mottram (1938) produced benign and malignant skin tumours in mice by the combined action of benzpyrene painting, insufficient on its own to produce warts, together with a single exposure of 800 to 2500 r units of beta radiation. In view of these findings, the safety of $I^{131}$ therapy for thyrotoxicosis must be questioned. However, a dosage sufficient to eliminate the thyroid, provided it is followed by maintenance with thyroxine, would presumably not be carcinogenic. The time factor is important ; it is likely that tumour formation would require a much longer time to be revealed in the human than in the rat.

I hestitate to join the vexed discussion of histological diagnosis of thyroid malignancy. But there is no doubt that the accepted criteria for other cancers do not always hold in the case of the thyroid. The frequent use in clinical medicine of the term " adenoma malignum " speaks for itself. Experimentally, Gorbman (1947) found hyperplastic thyroid tissue within the lumens of pulmonary vessels of chronically thiouracil treated mice as well as parenchymatous pulmonary deposits considered to be probably thyroid in origin. Yet a return to normal diet after prolonged goitrogen treatment was followed by involution of the supposedly cancerous thyroids. He considered that the hyperplastic thyroid tissue had entered the thyroid veins mechanically rather than by " malignant infiltration," and that the changes were entirely benign. The diagnostic significance of local venous tumour embolism is indeed difficult to assess in nodular goitres. One may assume, therefore, that two types of metastatic thyroid tissue exist, one, possibly resulting from a mechanical breakaway, still dependent upon thyrotrophic hormone for its survival, the other independent. Nevertheless, from the host's point of view the first type of metastasis may prove just as embarrassing as the second, so long as thyrotrophic hormone stimulation is maintained. And a lethal metastasizing tumour can reasonably be classified as malignant. The metastasizing prostatic tumour caused temporarily to regress by suppression of androgens essential for its maintenance might be regarded as an intermediate type of cancer. Prolonged thyroxine treatment, used in clinical medicine in the therapy of nodular goitre, may, by inhibiting thyrotrophic hormone production, not only shrink the goitre, but lessen the likelihood of mechanical production of thyroid metastases and of their survival.

### SUMMARY.

The carcinogenic potency on the thyroid of 32 μC. of $I^{131}$ was tested in a small series of rats alone, in combination with methylthiouracil, with acetylaminofluorene, and with the combined drugs. The radioactive iodine was found to significantly increase the formation of thyroid adenomas as compared with non $I^{131}$ treated controls in the following groups : those treated with $I^{131}$ alone, those treated with methylthiouracil and those treated with methylthiouracil plus acetylaminofluorene. One thyroid cancer was found in each of the latter two groups. The findings are discussed.

I am indebted to the U.S. Atomic Energy Commission for the supply of the isotope used, through the M.R.C. ; the British Empire Cancer Campaign for the acetylaminofluorene ; Dr. S. R. Pelc for the autoradiographs ; Mr. J. G. Griffin and Mr. L. J. Wright for the sections ; Mr. V. E. Wilmott for the photomicrographs ; Mrs. M. Donald and Mr. R. Rickwood for their care of the animals, and and S. Doniach for the calculations.

### REFERENCES.

Astwood, E. B., Sullivan, J., Bissell, A., and Tyslowitz, R.—(1943) *Endocrinol.*, **32**, 210.
Bielschowsky, F.—(1944) *Brit. J. exp. Path.*, **25**, 90.—(1945) *Ibid.*, **26**, 270.
Cox, A. J., Jun., Wilson, R. H., and De Eds, F.—(1947) *Cancer Res.*, **7**, 647.
Doniach, I., and Pelc, S. R.—(1949) *Proc. Roy. Soc. Med.*, **42**, 957.
*Iidem.*—(1950) *Brit. J. Radiol.*, **23**, 184.
Feller, D. D., Chaikoff, I. L., Taurog, A., and Jones, H. B.—(1949) *Endocrinol.*, **45**, 464.
Gorbman, A.—(1947) *Cancer Res.*, **7**, 746.
Griesbach, W. E., Kennedy, T. H., and Purves, H. D.—(1945) *Brit. J. exp. Path.*, **26**, 18.
Hall, W. H.—(1948) *Brit. J. Cancer*, **2**, 273.
*Idem* and Bielschowsky, F.—(1949) *Ibid.*, **4**, 534.
Laqueur, G. L.—(1949) *Cancer Res.*, **9**, 247.
Leblond, C. P., and Gross, J.—(1948) *Endocrinol.*, **43**, 306.
Mackenzie, C. G., and Mackenzie, J. B.—(1943) *Ibid.*, **32**, 185.
Money, W. L., and Rawson, R. W.—(1947) *Trans. Amer. Ass. Goitre*, 171.
Mottram, J. C.—(1938) *Amer. J. Cancer*, **32**, 76.
Purves, H. D., and Griesbach, W. E.—(1946) *Brit. J. exp. Path.*, **27**, 294.—(1947) *Ibid.*, **28**, 46.
Skanse, B. N.—(1948) *J. clin. Endocrinol.*, **8**, 707.